# Evaluation the interaction of ABC multidrug transporter MDR1 with thymoquinone: substrate or inhibitor?

**DOI:** 10.22038/ijbms.2020.44216.10381

**Published:** 2020-10

**Authors:** Vahideh Keyvani, Zeinab Nasserifar, Mohammad-Reza Saberi, Seyed Ahmad Mohajeri, Sepideh Arabzadeh, Farajollah Shahriari Ahmadi, Hossein Hosseinzadeh, Seyedeh Mahya Shariat Razavi, Fatemeh Kalalinia

**Affiliations:** 1 Department of Biology, Faculty of Science, Shahid Chamran University of Ahvaz, Ahvaz, Iran; 2 Department of Biotechnology and Plant Breeding, Faculty of Agriculture, Ferdowsi University of Mashhad, Mashhad, Iran; 3 Pharmaceutical Research Center, Pharmaceutical Technology Institute, Mashhad University of Medical Sciences, Mashhad, Iran; 4 Biotechnology Research Center, Pharmaceutical Technology Institute, Mashhad University of Medical Sciences, Mashhad, Iran; 5 Department of Biology, Faculty of Sciences, University of Sistan and Baluchestan, Zahedan, Iran

**Keywords:** Drug transporters, MDR1, Multi drug resistance, Nigella sativa, Thymoquinone

## Abstract

**Objective(s):**

Thymoquinone (TQ) has valuable medical properties like anticancer effects. Development of multidrug resistance (MDR) phenotype is one of the most important factors in failure of cancer chemotherapy. The aim of this study was to evaluate the mode of interaction of TQ and MDR1, a major MDR-related protein in gastric cancer drug resistant EPG85-257RDB cells, and its parental non-resistant EPG85-257 cells.

**Materials and Methods:**

MTT assay was used to assess the effects of TQ and doxorubicin (DOX) on cell viability of tested cell lines and TQ effect on pump performance. HPLC analyses were used to measure the input and output of TQ in EPG85-257RDB cells. Molecular docking studies were used to identify interactions between TQ and MDR1.

**Results:**

TQ inhibited cell viability in a time and concentration-dependent manner. Co-treatment of the cells with TQ and DOX did not significantly affect the amount of cell viability in comparison with DOX treatment alone. The HPLC analyses showed that more than 90% of TQ entered to EPG85-257RDB during 1 hr of treatment with TQ, but it was unable to exit from the cells. Moreover, there was no difference between influx and efflux amount of TQ in cells with inhibited and non-inhibited MDR1 transporters. Molecular docking studies revealed that TQ had a higher inhibitory constant to bind to active site of MDR1 protein as compared to specific inhibitor (verapamil) and substrate (vinblastine) of this transporter.

**Conclusion:**

These results proposed that TQ does not work as an inhibitor or a substrate of MDR1 transporter.

## Introduction

Cancer has become one of the most common causes of mortality and morbidity in societies in recent years. Various chemotherapy medications are considered as common cancer treatment, but unfortunately resistance to chemotherapy commonly causes chemotherapy failure in cancer patients. One of the most important reasons for the resistance to chemotherapy is the multidrug resistance (MDR) phenomenon (1). The presence of ATP Binding Cassette Transporters (ABC transporters) is an important mechanism that causes resistance to chemotherapy. ABC transporters actively transport the cytotoxic substances out of cancerous cells and keep intracellular concentrations of these substances below cytotoxic levels (2, 3). Nowadays, MDR1 is considered to be the most famous transporter involved in drug resistance in various cancer types. In an idealistic view, it is important to find new cytotoxic drugs and herbal compounds by the way that not only they would not be affected by ABC transporters involved in multiple drug resistance, but also they could be able to control these transporters. 

*Nigella sativa*, an annual flowering plant of the *Ranunculaceae* family, is native to south and southwest Asia. The effects of this plant’s extract on the treatment of many diseases such as cancer, hypertension, asthma, and diabetes have been proven in several studies (4). Evidence from various studies suggests the use of thymoquinone (TQ), the active ingredient of *N. sativa* extract, alone or in combination with other drugs for cancer treatment based on its important role in apoptosis in cancer cells (5), inhibiting metastasis (6) and increasing the effectiveness of other anticancer drugs (7-9). In addition, there are evidences of the relationship between TQ and regulation of the expression of ABC proteins such as the effect of TQ on multidrug resistance-associated proteins (MRPs) in rats treated with cisplatin (10, 11). There was no detailed study on the possible role of TQ in controlling the drug resistance phenomenon. In this study, the reaction between TQ and MDR1 in EPG85-257RDB gastric cancer cell line, that is resistant to doxorubicin via highly expression of the MDR1, and its parental non-resistant EPG85-257 cells were evaluated. 

## Materials and Methods

***Materials***

Fetal bovine serum (FBS) and RPMI 1640 were purchased from Gibco (USA). Trypan blue, Methanol, KCl, NaCl, NaHCO_3_, Na_2_HPO_4_, and K_2_HPO_4_ were obtained from Merck (Germany). TQ, dimethyl sulfoxide (DMSO) and 3-(4,5-dimethylthiazol-2-yl)-2,5-diphenyl tetrazolium bromide (MTT) were achieved from Sigma-Aldrich (Germany). Gastric cancer parental and resistant cell lines were generously provided by Prof. Herman Lage (Molecular pathology department, Charite Campus Mitte, Berlin, Germany). The cells were cultured in complete culture medium (RPMI 1640 contained FBS 10% (v/v)) at 37 ^°^C in humidified air containing 5% CO_2_. 

***Preparation of the TQ and doxorubicin solutions***

TQ was dissolved in DMSO and phosphate-buffered saline (PBS) to a final concentration of 10 mM and stored at −20 ^°^C. Also, doxorubicin with an initial concentration of 3440 µM was diluted to 5 µM concentration with complete culture medium. For each experiment, these drugs were freshly diluted to the final concentrations of 10-100 µM for TQ and 0-500 nM for doxorubicin.

***Evaluation of the EPG85-257 and EPG85-257RDB cells viability with MTT cytotoxicity assay***

Drug sensitivity of the EPG85-257 and drug-resistant EPG85-257RDB cell lines to TQ and doxorubicin drugs were evaluated by MTT assay. Cells were seeded at an initial density of 10^4^ cells/well in 96-well plates in a volume of 100 μl. The plates were incubated at 37 ^°^C in a 5% CO_2_-supplemented atmosphere for 24 hr. Subconfluent cells were treated with TQ (0–100 µM) or doxorubicin (0-500 nM) drugs in the final volume of 100 μl of complete growth medium in each well. The solvent control wells had DMSO in the growth medium at the equal volumes to those used for the test compounds. Cell viability was measured after 24–72 hr by MTT assay. The reduced MTT dye was solubilized with DMSO (100 μl/well) and absorbance was determined by an ELISA plate reader (BioTek, Germany) with a test wave length of 550 nm and a reference wavelength of 630 nm. Each experiment was performed in triplicate and was repeated at least three times. The percentage of cell proliferation was calculated using the ratio of OD_test_/OD_control _×100

In next step, EPG85-257 and EPG85-257RDB cells were cultured in the presence or absence of non-toxic concentrations of TQ (0.5-20 µM), and various concentrations of doxorubicin (0-500 nM) to evaluate the effects of TQ on the doxorubicin cytotoxicity.

***Evaluation of the TQ influx and efflux through EPG85-257RDB cell line by HPLC analyses***

Preparation of the standard curve of TQ in methanol medium (HPLC grade from Duksan, Korea) or RPMI 1640 medium was performed by HPLC analysis (9000Younglin Acme system, South Korea) using a C18 ODSA column (4.6 × 150 mm, 5 μm) at 30 ^°^C. The mobile phase was a mixture of water, methanol and isopropanol at a ratio of 50:45:5 and the flow rate was 1 ml/min. Data analysis was performed by Autochro-3000 software and through isocratic method. The eluent was monitored by a UV array detector at 254 nm. Each sample was analyzed in triplicate. 

Evaluation of the amount of TQ that influxed into EPG85-257RDB cells and also the amount of TQ that effluxed from EPG85-257RDB cells into the culture medium by MDR1 transporter were measured. For this purpose, EPG85-257RDB cells were seeded at density of 5 ×10^5^ cells/well in 6-well plates in a volume of 2 ml and the plates were incubated at 37 ^°^C for 24 hr. Subconfluent cells were washed two times with PBS to completely remove FBS and then treated with 50 or 100 µM of TQ in the presence or absence of verapamil 100 µM (specific inhibitor of MDR1) for one hour in a culture medium without FBS at 37 ^°^C in the presence of CO_2_ 5%. The control samples were treated with TQ or verapamil alone. In this step, the supernatant was removed to evaluate the amount of TQ entry into EPG85-257RDB cells (influx) and the cells were treated for additional one and four hours with culture medium without TQ and FBS in the presence or absence of verapamil for efflux assay. Finally, for each influx or efflux assay, the amount of TQ present in the supernatant of the cells was determined by HPLC using the standard curve. The same concentrations of TQ in a culture medium without cell were analyzed by HPLC to achieve the theoretical amount of TQ. Finally, the amount of TQ influx was calculated as: theoretical amount of TQ – total amount of TQ in supernatant.

In efflux assay, because the released amount of TQ was very low and could not be detected by the HPLC device, the “standard additions method” was used to solve the problem. In this method, specific concentrations of compound under study are added to the desired samples and then analyzed by HPLC. Since the added compound at each concentrations have a certain value, the unknown amount of the compound under study can be calculated. In this study, a sample of 300 µl was taken from supernatant after one and four hours of incubation. Next, the sample was divided into three microtubes (100 µl each). Subsequently, based on standard additions method, 100 µl of TQ solutions containing various concentrations of 30, 60 and 80 µM was added to each microtube and the peak area of TQ was determined by HPLC.

***Molecular docking studies for determination the connection type between TQ and MDR1***

Chem bio Draw 8.0 was used (Cambridge soft, 2003) to prepare the structure of vinblastine, verapamil and TQ molecules. Molecular docking of these molecules was carried out using the MGLTools and AutoDock Tools 4 (AD4) software packages and the Lamarckian genetic algorithm (LGA) for the prediction of binding affinity and searching for the optimum binding site. Also, the AutoDock Tool (ADT) was employed to set up and perform blind docking calculations. Polar hydrogen was added using the Hydrogen module in AutoDock Tools (ADT). After that, Kollman united atom partial charges were assigned for the receptor. Docking between the TQ and MDR1 pump in the active site of the protein was performed to determine the interaction between the MDR1 protein and TQ, the role of TQ in the active site of the protein, and comparison of its inhibitory constant with the substrate and inhibitor of MDR1.

***Statistical analysis***

Results (mean±SD) were reported based on three independent experiments. Statistical analyses were performed by SPSS version 16 using ANOVA, with the Tukey’s *post hoc* to show significant differences between the data. *P-*values<0.05 were considered significant.

## Results

***Effect of TQ on the proliferation rate of EPG85-257 and EPG85-257RDB cancer cell lines***

**Figure 1:**
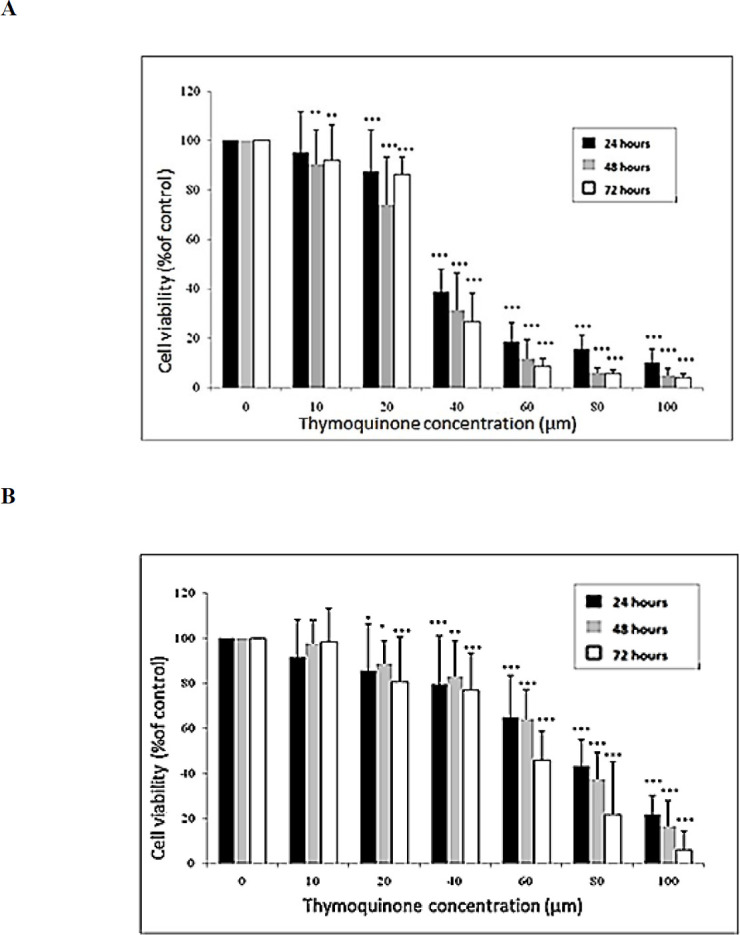
The effects of thymoquinone (TQ) on the cell viability of EPG85-257 (a) and EPG85-257RDB (b) cell lines. The cells were incubated with various concentrations of TQ at 37 ^°^C for 24, 48 and 72 hr. Cell viability was measured by the MTT assay. Each experiment was repeated independently three times in triplicate tests and data are shown as mean±SD. *P*-value<0.05 with *, *P*-value≤0.01 with ** and *P*-value ≤0.001 with ***

To investigate the effects of TQ on the cell survival, gastric cancer cells were incubated in the presence or absence of various concentrations of TQ (0–100 µM) for 24, 48 and 72 hr, then subjected to MTT cytotoxicity assay. TQ showed inhibitory effects on the cell growth rate of EPG85-257 cells in a concentration and time-dependent manner (Figure 1a). TQ exhibited a similar inhibitory pattern in EPG85-257RDB (Figure 1b). The IC_50_ values (Table 1) revealed that the effect of TQ on the inhibition of EPG85-257 cells growth is more than EPG85-257RDB.

***Effect of doxorubicin on the proliferation rate of EPG85-257 and EPG85-257RDB cancer cell lines***

**Figure 2:**
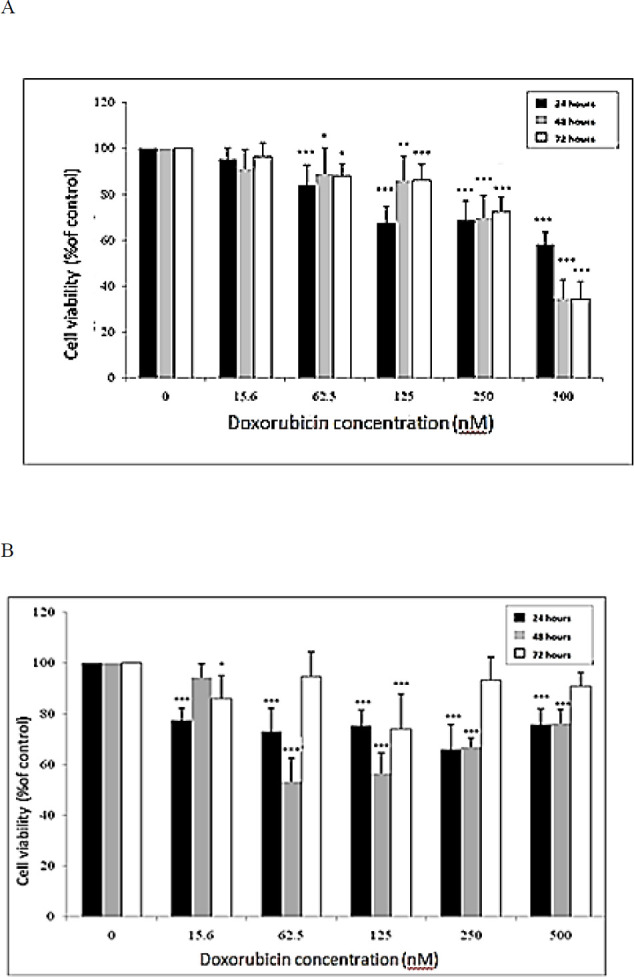
The effects of doxorubicin (Dox) on cell viability of EPG85-257 (a) and EPG85-257RDB (b) cell lines. The cells were incubated with various concentrations of doxorubicin at 37 ^°^C for 24, 48 and 72 hr. Cell viability was measured by the MTT assay. Each experiment was repeated independently three times in triplicate tests and data are shown as mean±SD. 0.01≥ *P*-value<0.05 with *, 0.001<*P*-value ≤0.01 with ** and *P*-value ≤0.001 with ***

Effects of doxorubicin were investigated on the cell survival of EPG85-257 and EPG85-257RDB. Cells were incubated in the presence or absence of various concentrations of doxorubicin (0–500 nM) for 24, 48 and 72 hr and then subjected to MTT cytotoxicity assay as shown in Figure 2. Doxorubicin showed inhibitory effects on the growth rate of EPG85-257 cells in a concentration and time-dependent manner. It exhibited an inhibitory effect on the EPG85-257RDB cell growth rate with lower potency than it had on the parental non-resistant cell line.

***Simultaneous effect of doxorubicin and TQ on the proliferation rate of EPG85-257 and EPG85-257RDB cancer cell lines***

To test the combining effects of TQ and doxorubicin on the survival of EPG85-257 and EPG85-257RDB cancer cell lines, the effects of 30 different combinations (0–20 μM of TQ with 0–500 nM of doxorubicin) were evaluated by MTT assay. The combinatorial effects on cell survival were analyzed after 24, 48, and 72 hr of incubation. There were no significant differences in EPG85-257 and EPG85-257RDB cancer cell lines viabilities between all TQ + doxorubicin concentrations and their controls with the equal amount of doxorubicin (Table 2). 

***The results of HPLC analysis for evaluation of the TQ influx or efflux through EPG85-257RDB cell line***

**Figure 3:**
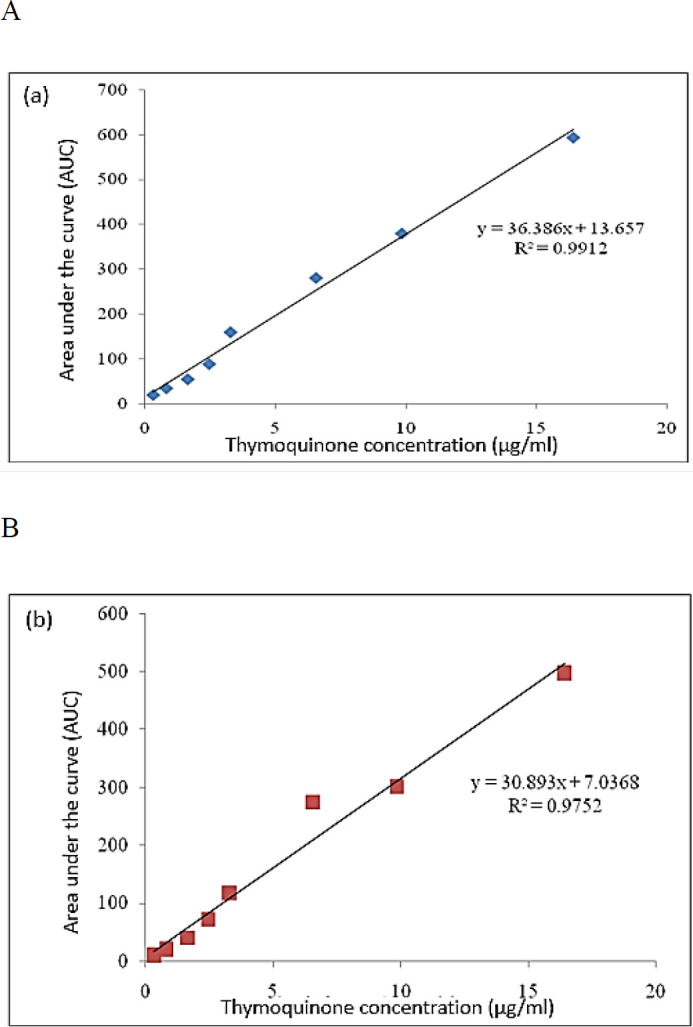
Standard curve of thymoquinone (TQ) in methanolic medium (a) and RPMI 1640 medium (b)

HPLC analysis (C18 column, UV 730 detector, mobile phase flow rate: 1 ml/min, mobile phase with 50% water, 45% methanol and 5% isopropanol, solvent transfer module SP930D, solvent vacuum mixer degasser SDV50A) was used to determine whether TQ could be a substrate for the MDR1 transporter or not. At first, the standard curves for TQ were generated and used for evaluation of the amount of TQ influx or efflux through the EPG85-257RDB cell. For this purpose, different concentrations of TQ (0-20 µg/ml) in methanolic or RPMI 1640 medium were prepared and injected into the HPLC device and the standard curve was plotted using the amounts of area under the curve at different concentrations of TQ. Each standard was run with five repetitions (Figure 3). 

In influx assay, EPG85-257RDB cells were treated with TQ 50 or 100 µM in the presence or absence of verapamil 100 µM (specific inhibitor of MDR1) for one hour in a culture medium without FBS, and the amount of TQ present in the supernatant of the cells was determined by HPLC. The results showed that there was no significant difference in amount of intracellular accumulation of TQ between samples that had been treated with TQ alone or TQ combined with verapamil (data not shown).

**Figure 4:**
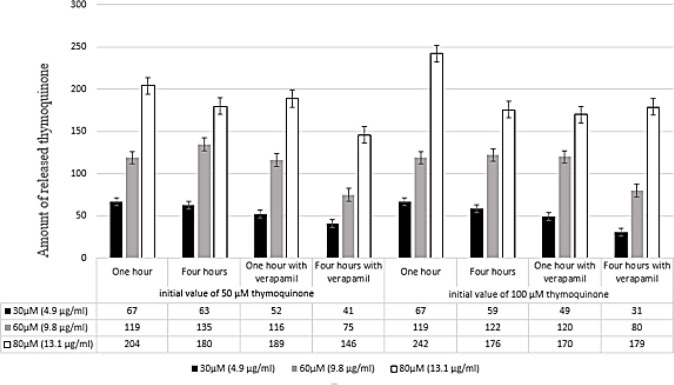
Results of the standard addition method of thymoquinone (TQ) in the EPG85-257RDB cell culture medium. The cells were incubated with various concentrations of TQ (30, 60, and 80 µM) at 37 ^°^C for 1 and 4 hr in presence or absence of verapamil. The additional standard method was used to evaluate of the amount of TQ exiting from EPG85-257RDB cells through a MDR1 transporter. Each experiment was repeated independently three times in triplicate tests and data are shown as mean±SD. 0.01≥ *P*-value<0.05 with *, 0.001<*P*-value ≤0.01 with ** and *P*-value ≤0.001 with ***

In efflux assay, at first EPG85-257RDB cells were treated with TQ 50 or 100 µM in the presence or absence of verapamil 100 µM (specific inhibitor of MDR1) for one hour (accumulation time). Then, the supernatant was removed and the cells were washed with PBS and treated with culture medium without TQ and FBS in the presence or absence of verapamil. After one and four hours of incubation, the released amount of TQ into the supernatant was detected by the HPLC device using the standard additions method (Figure 4). The results exhibited that, in the cell containing TQ-verapamil (the specific transporter inhibitor) that was considered as a control in the test, no TQ exiting was observed. Also, there was no significant difference in amount of TQ exiting between samples that had been treated with TQ alone or TQ combined with verapamil. 

***The results of molecular docking studies for investigation of the interaction between the MDR1 protein with TQ***

Molecular docking was used to study the interaction between the MDR1 protein and TQ to determine the role of TQ against this transporter. For this purpose, the interaction between MDR1 protein and TQ was compared to interaction with its specific inhibitor (verapamil) and its substrate (vinblastine). One hundred bindings of verapamil, TQ, and vinblastine molecules were investigated in the active site of protein, and the best result was determined in terms of K_i _(protein inhibition constant). The best K_i_ values were 3.3 µM with a binding affinity of -6.48 Kcal/mol for verapamil, 35.42 M with a binding affinity of -6.07 Kcal/mol for TQ and 24.7 nM with a binding affinity of -10.38 Kcal/mol for vinblastine. 

**Table 1:** The IC_50_ values of thymoquinone (TQ) against EPG85-257 parental and resistant cells

Cell line	Time (hr)	IC_50_ (µM)
EPG85-257	24	46
	48	40
	72	39
EPG85-257RDB	24	70
	48	68
	72	55

**Table 2:** The effects of different concentrations of thymoquinone on the cell survival percentages of EPG85-257 and EPG85-257RDB cell line under treatment with doxorubicin (mean±SEM)

Cell line	Time (hr)	Dox(nM)	Thymoquinone (µM)			
EPG85-257			0	5	10	20
	24	0	100 ± 0.00	94.61 ± 2.68	98.01 ± 1.85	89.94 ± 2.27
		15	95.23 ± 1.46	75.98 ± 3.07	93.87 ± 3.76	88.69 ± 3.47
		62	83.94 ± 2.51	73.43 ± 3.07	77.54 ± 4.41	79.74 ± 3.41
		125	67.69 ± 2.08	61.46 ± 3.01	63.60 ± 4.2	69.43 ± 3.8
		250	68.68 ± 2.45	61.54 ± 2.26	74.69 ± 3.12	72.51 ± 3.27
		500	58.28 ± 1.48	59.15 ± 2.54	59.49 ± 3.47	58.46 ± 2.3
	48	0	100 ± 0.00	92.91 ± 2.18	88.98 ± 1.51	91.44 ± 2.28
		15	91.11 ± 2.41	89.85 ± 2.06	86.18 ± 2.36	92.95 ± 5.49
		62	88.94 ± 2.79	82.65 ± 2.86	77.67 ± 4.63	85.82 ± 5.04
		125	86.035 ± 3.01	76.48 ± 2.44	83.83 ± 6.46	83.86 ± 5.02
		250	69.75 ± 2.79	70.04 ± 2.2	66.08 ± 2.79	74.77 ± 4.83
		500	34.51 ± 2.39	34.56 ± 3.66	38.71 ± 1.99	34.37 ± 3.47
	72	0	100 ± 0.00	92.46 ± 2.43	93.01 ± 2.54	97.06 ± 1.93
		15	96.33 ± 1.71	89.56 ± 2.05	84.54 ± 2.82	102.76 ± 1.43
		62	87.96 ± 1.49	78.35 ± 2.85	79.18 ± 2.93	90.43 ± 3.14
		125	86.21 ± 2.03	76.05 ± 2.76	70.9 ± 2.52	88.73 ± 2.83
		250	72.45 ± 1.78	77.61 ± 4.63	79.12 ± 2.63	85.47 ± 1.06
		500	34.39 ± 2.15	30.32 ± 0.99	22.08 ± 1.37	19.01 ±1.96
EPG85-257RDB	24	0	100 ± 0.00	90.45 ± 1.68	93.30 ± 5.49	93.03 ± 7.3
		15	77.43 ± 1.61	81.24 ± 2.00	79.02 ± 4.45	87.13 ± 5.45
		62	73.02 ± 3.06	76.13 ± 2.63	81.27 ± 3.09	75.51 ± 3.89
		125	75.22 ± 2.10	75.57 ± 2.30	67.43 ± 1.76	78.17 ± 3.56
		250	65.90 ± 3.32	73.71 ± 1.58	76.99 ± 5.76	80.69 ± 2.89
		500	75.81 ± 2.01	72.32 ± 1.87	72.75 ± 3.59	83.53 ± 2.75
	48	0	100.00 ± 0.00	98.56 ± 1.33	93.00 ± 2.04	89.31 ± 1.90
		15	94.35 ± 1.81	74.79 ± 4.02	71.29 ± 2.63	75.01 ± 3.81
		62	53.27 ± 3.08	46.90 ± 2.15	47.27 ± 1.07	79.22 ± 4.68
		125	56.49 ± 2.69	46.78 ± 4.22	62.11 ± 4.45	52.94 ± 1.91
		250	66.93 ± 1.17	62.67 ± 0.87	39.37 ± 1.50	68.05 ± 2.81
		500	76.17 ± 1.82	68.09 ± 1.73	64.71 ± 5.01	65.71 ± 2.57
	72	0	100.00 ± 0.00	97.81 ± 2.10	92.36 ± 1.04	95.74 ± 4.85
		15	85.92 ± 3.06	95.45 ± 4.84	94.34 ± 5.15	99.44 ± 5.08
		62	94.81 ± 3.21	85.85 ± 2.88	81.23 ± 5.23	93.13 ± 7.90
		125	73.95 ± 4.60	87.29 ± 4.18	96.58 ± 5.95	102.15 ± 3.30
		250	93.23 ± 3.01	92.27 ± 2.97	96.99 ± 6.58	97.89 ± 2.70
		500	98.80 ± 3.62	99.27 ± 2.39	96.09 ± 1.51	96.31 ± 1.67

Note: The results of the LSD test, which compared the effects of all thymoquinone (TQ) concentrations on the toxicity of each of the various concentrations of doxorubicin (Dox) during 24, 48, and 72 hr. *, *P*-value<0.05; **,* P*-value<0.01; ***, *P*-value<0.001

## Discussion

MDR1 is one of the most important reasons for the insufficient effectiveness of chemotherapy drugs in cancer treatment. Therefore, finding new anticancer drugs that are not substrate for this transporter and act as its inhibitors would be very valuable (12, 13). Various studies proved the effects of TQ, one of the main ingredients of *N. sativa* extract, on the treatment of many diseases including cancers (6, 14, 15). In this study, mode of interaction between TQ and MDR1 was evaluated in EPG85-257RDB gastric cancer cell lines that are resistant to doxorubicin and its parental non-resistant EPG85-257 cells. 

The results of this study showed that TQ alone and doxorubicin alone significantly decreased the viability of gastric cancer cells, while there were no significant differences in cancer cell lines viabilities between all co-treatment of TQ + doxorubicin concentrations and controls with the equal amount of doxorubicin alone. In a similar study, the effects of TQ and doxorubicin on the viability of several cancer cell lines were investigated. Their results showed that TQ and doxorubicin reduced cell survival in these cell lines. IC_50_ values varied from one cell to another one and ranged from 18 to 57 μM for TQ and 0.05 to 18.3 μM for doxorubicin (9). Equitoxic combination of TQ and cisplatin showed additive to synergistic interaction against human oral squamous cell carcinoma cell (UMSCC-14C) and normal oral epithelial cells (OEC) (16). 

In this study, the resistant cells (EPG85-257RDB) showed less sensitivity to the toxicity effects of TQ and doxorubicin in comparison with the parental non-resistant cells (EPG85-257). Previously, it has been shown that the expression of MDR1 in the daunorubicin-selected subline EPG85-257RDB is significantly higher than its expression in EPG85-257 cells (17). Therefore, it seems that MDR1 could transport TQ and doxorubicin out of the EPG85-257RDB cells more than EPG85-257, resulting in their less cytotoxicity in resistance cell line. The question remains that whether TQ could be used to overcome the MDR phenotype. In this study, the cytotoxicity of various mixtures of TQ and doxorubicin on the parental non-resistant or drug resistant EPG85-257 was evaluated. The results showed that the exposure of EPG85-257 and EPG85-257RDB cells to TQ and doxorubicin simultaneously did not significantly change the toxicity of doxorubicin in each cell line. Therefore, it can be concluded that TQ was unable to inhibit the MDR1 transporter and increase the intracellular accumulation of doxorubicin in tested cell lines. 

Molecular docking studies showed that the TQ affinity to the MDR1 transporter is much lower than the substrate (vinblastine) and specific inhibitor (verapamil) of this transporter. The results of binding energy and the inhibitory constant showed a significant increase regarding TQ composition, which proposed that TQ does not compete with vinblastine and verapamil in occupying active sites of protein (the binding energy and the inhibitory constant of the vinblastine, verapamil and TQ were -10.38 Kcal/mol and 24.7 nM, -6.48 Kcal/mol and 3.53 μM and -6.07 Kcal/mol and 35.42 μM, respectively). In fact, TQ is not able to occupy the active site of the protein and inhibit the MDR1 transporter.

The amount of TQ that influxed into EPG85-257RDB cells and also the amount of TQ that effluxed into the cell culture medium by MDR1 transporter were measured by HPLC. The results of HPLC analysis showed no differences in the rate of entry of TQ into the cells in presence or absence of verapamil. Based on these results, there are two hypotheses: first, the TQ is not a substrate for the MDR1 transporter and is not transported to the extracellular medium (if the TQ is a substrate of the transporter, it was exported out of the cell through this transporter based on MDR phenomenon, and its peak could be detected by HPLC). In the second hypothesis, it may be supposed that after entering the cell, the TQ may interfere with the proteins within the cell and could not exit from the cell. This phenomenon is probably similar to the formation of complexes between TQ and the protein present in the culture medium and precipitation of this complex at the stages of samples preparation. In these cases, it was observed that if the complete culture medium containing FBS was used to prepare the TQ samples to draw the calibration curve, the TQ peak would not be detectable. In various studies that investigated TQ by HPLC analyses, the analyses were performed in an *in vitro* medium containing isopropanol and methanol (18, 19). None of these studies have been conducted in an *in vitro* medium containing proteins and cell culture media. 

## Conclusion

In this study, we pointed to evaluate the mode of interaction of TQ and MDR1 in gastric cancer drug resistant cell line EPG85-257RDB in comparison with its parental non-resistant cell line EPG85-257. The results revealed that TQ inhibited cell viability in a time and concentration-dependent manner, while co-treatment of the cells with TQ and DOX did not significantly affect the amount of cell viability in comparison with DOX treatment alone. Also, due to the higher expression of MDR1 transporter in the EPG85-257RDB cell, the transport of TQ and doxorubicin in the EPG85-257RDB cell is higher than the EPG85-257 parent cell and toxicity of these drugs is lower in the EPG85-257RDB cell. The results of the HPLC assay did not show any difference in the rate of influx or efflux of TQ into the cells in presence or absence of verapamil (specific inhibitor of MDR1). Also, empirical observations of molecular docking studies showed that the tendency of MDR1 to its substrate and its specific inhibitor is much more than TQ. Furthermore, TQ has not ability to compete with vinblastine and verapamil in occupying the active site of the MDR1 protein. Finally, this study proposed that TQ does not act as inhibitor or substrate for MDR1 transporter.
